# Ambulatory end-stage liver disease in Ghana; patient profile and utility of alpha fetoprotein and aspartate aminotransferase: platelet ratio index

**DOI:** 10.1186/s12876-020-01581-9

**Published:** 2020-12-26

**Authors:** Yvonne Ayerki Nartey, Yaw Asante Awuku, Adwoa Agyei-Nkansah, Amoako Duah, Sally Afua Bampoh, Joshua Ayawin, Shadrack Osei Asibey, Niklas K. Björkström, Weimin Ye, Mary Yeboah Afihene, Lewis Rowland Roberts, Amelie Plymoth

**Affiliations:** 1 Department of Medical Epidemiology and Biostatistics, Karolinska Institutet, Nobels väg 12A, 17177 Stockholm, Sweden; 2 Department of Internal Medicine, School of Medical Sciences, University of Cape Coast, Cape Coast, Ghana; 3 Department of Medicine and Therapeutics, University of Ghana Medical School and Korle Bu Teaching Hospital, Accra, Ghana; 4 Department of Medicine, St. Dominic Hospital, Akwatia, Ghana; 5 Department of Medicine, Greater Accra Regional Hospital, Accra, Ghana; 6 Department of Medicine, Komfo-Anokye Teaching Hospital, Kumasi, Ghana; 7 Catholic University College, Sunyani, Ghana; 8 Department of Medicine Huddinge, Center for Infectious Medicine, Karolinska Institutet, Karolinska University Hospital, Stockholm, Sweden; 9 Department of Medicine, Kwame Nkrumah University of Science and Technology, Kumasi, Ghana; 10 Division of Gastroenterology and Hepatology, Mayo Clinic, Rochester, MN USA

**Keywords:** Liver cirrhosis, Hepatocellular carcinoma, End-stage liver disease, AST to platelet ratio index (APRI) score, Alpha fetoprotein (AFP), Ghana, Sub-Saharan Africa

## Abstract

**Background:**

End-stage liver disease (ESLD) is a major burden on public health, particularly in sub-Saharan Africa, where hepatitis B virus (HBV) is an important risk factor. We aimed to describe clinical characteristics of ESLD from cirrhosis or hepatocellular carcinoma (HCC) and the performance of aspartate aminotransferase (AST)—platelet ratio index (APRI) and alpha fetoprotein (AFP) in Ghana.

**Methods:**

We performed an observational cross-sectional study in outpatient hepatology clinics at three teaching hospitals in Ghana, West Africa. One hundred and forty-one HCC, 216 cirrhosis and 218 chronic HBV patients were recruited by convenience sampling. Sociodemographic, history and examination, laboratory, and disease staging information were shown using descriptive statistics. Performance of the APRI score in diagnosis of cirrhosis and AFP in the diagnosis of HCC was determined using AUROC analysis.

**Results:**

Median age at presentation was 44 years for HCC and 46 years for cirrhosis. HBV was found in 69.5% of HCC and 47.2% of cirrhosis cases, and HCV in 6.4% and 3.7% respectively. APRI cut-off of 2 had sensitivity of 45.4% and specificity of 95% in diagnosis of cirrhosis, and cut-off of 1 had sensitivity of 75.9% and specificity of 89%. AUC of AFP was 0.88 (95% CI 0.81–0.94) in diagnosis of HCC. Low monthly income was associated with lower odds of undertaking AFP. Thirty one percent of cirrhotic persons were Child–Pugh C, and 67.9% of HCC patients had advanced or terminal disease at presentation.

**Conclusions:**

Our findings emphasize the young age of ESLD patients in Ghana and the advanced nature at presentation. It highlights shortcomings in surveillance and the need for policies to address the burden and improve outcomes in Ghana.

## Background

Decompensated liver cirrhosis and/or liver cancer, collectively termed end-stage liver disease (ESLD) [1] are often the final stage of long-standing liver disease, and are responsible for a significant burden of morbidity and mortality worldwide. It is estimated that globally, over 1.2 million deaths in 2016 were as a result of liver cirrhosis alone [2]. It is probable that cirrhosis accounts for even more deaths, since there is a lack of reliable data from many countries in sub-Saharan Africa [3, 4]. The incidence and mortality of liver cancer show similar trends, since patients often get their diagnosis at an advanced stage. In 2016 alone, there were 1 million incident cases of liver cancer and 829,000 deaths worldwide. In Western sub-Saharan Africa, liver cancer is the leading cause of incident cancer cases and has the highest cancer related mortality [5].

Risk factors for disease vary between different parts of the world. In the Western world, the major risk factors for liver cirrhosis and the main type of liver cancer, hepatocellular carcinoma (HCC), are chronic hepatitis C virus (HCV) infection, alcoholic liver disease, and non-alcoholic fatty liver disease, the latter of which is associated with metabolic disorders such as obesity and type 2 diabetes [6]. However, in Sub-Saharan Africa, where the prevalence of ESLD has continued to increase, the main risk factor is chronic hepatitis B virus (HBV) infection [7]. In West Africa, exposure to environmental toxins such as dietary aflatoxins, and the use of oral herbal medication are also thought to increase the burden of chronic liver disease [4, 8].

Previous studies on the characteristics and clinical profile of ESLD patients in sub-Saharan Africa have primarily focused on liver cancer and have recognized limitations in data collection due to challenges in retrieval of records, compounded by the fact that they have been retrospective in design [9, 10]. Furthermore, in Ghana, there are few studies on the characteristics and clinical profile of patients presenting with ESLD. It is thought that similar to liver cancer, most patients with cirrhosis in Ghana present late [11], however there is little in the published literature to describe the stage and severity at presentation, nor the common causes of ESLD in Ghana. Additionally, little is known about the performance of non-invasive markers for diagnosis of ESLD in this region. This information is crucial for the recognition of the extent of the liver disease burden by policy makers and advocates, and the development of strategies for improved surveillance and management of patients with liver disease. Thus, the aims of this study were to describe the clinical characteristics of ESLD patients in Ghana, and to assess the utility of the aspartate aminotransferase (AST)—platelet ratio index (APRI) score and alpha fetoprotein (AFP) in diagnosis of cirrhosis and HCC among this cohort.

## Methods

### Study design and patients

**Fig. 1:**
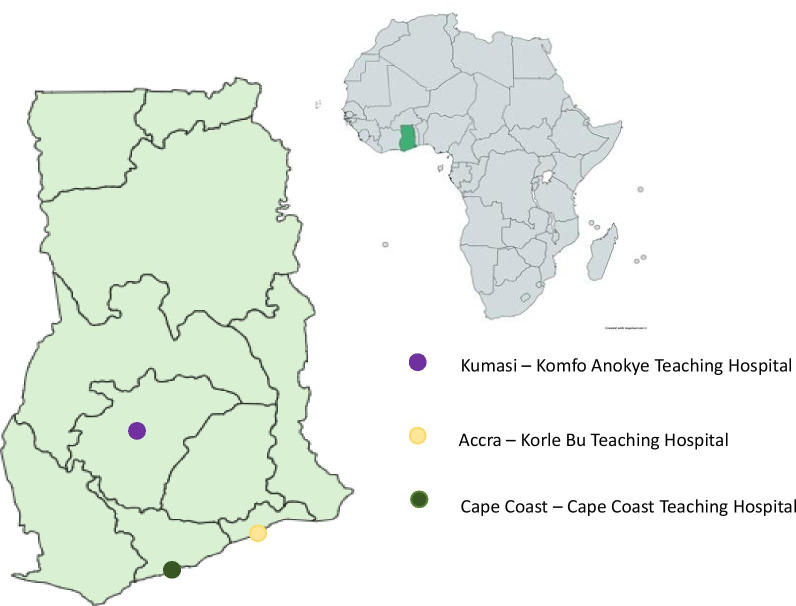
Map of Ghana showing location of sites involved in study (Created with mapchart.net)

We performed a multi-center outpatient clinic based cross-sectional study in Ghana. Liver disease patients attending hepatology clinics at the three largest teaching hospitals in Ghana—Korle Bu Teaching Hospital in Accra, Komfo Anokye Teaching Hospital in Kumasi, and Cape Coast Teaching Hospital in Cape Coast (Fig. 1) were recruited by convenience sampling for the study. These hospitals were chosen because they are tertiary referral centers that hold weekly specialist hepatology clinics and thus serve referred patients from the densely populated southern half of Ghana, from a wide range of socioeconomic backgrounds. Additionally, these teaching hospitals are the main referral centers for patients seen in private healthcare facilities across the country who require specialty care. They therefore serve as a bridge between public and private healthcare in the country and reflect the profile of ambulatory ESLD patients seen in allopathic healthcare centers in Ghana.

All patients referred to the hepatology clinics of these three teaching hospitals with liver cirrhosis, hepatocellular carcinoma or chronic HBV infection, and in whom the diagnosis was subsequently confirmed based on clinical, serological, and radiological evidence formed the target population from which the study population was obtained. Convenience sampling was used to recruit a total of 575 participants who fitted inclusion and exclusion criteria and who gave informed consent, in order to obtain a large sample of ESLD and chronic HBV patients. The inclusion criteria for ESLD patients were: (1) age above 18 years; and (2) diagnosed with liver cirrhosis or primary HCC based on clinical, serological, and/or radiological evidence. Chronic HBV patients were diagnosed based on serologic evidence and no evidence of liver cirrhosis following a negative liver ultrasound. Criteria for exclusion from the study were: (1) insufficient evidence for diagnosis.

### Data collection

Data collection took place between January 2017 and December 2018. Clinical data was obtained through patient medical records and included personal and family history of liver disease or viral hepatitis, history of alcohol consumption, and the presence of co-morbidities such as type 2 diabetes mellitus. Clinical examination for features such as ascites and hepatic encephalopathy were also performed. Additionally, investigations related to a diagnosis of liver diseases were obtained. These included complete blood count, including platelet count, liver chemistries and tests of liver function; viral serologies including hepatitis B surface antigen (HBsAg), antibodies to hepatitis C virus (anti-HCV), and antibodies to human immunodeficiency virus (HIV); and autoantibody tests including antinuclear antibody, anti-smooth muscle antibody, and any other relevant autoantibody tests. Additional information collected included HBV DNA quantification and AFP in patients who could afford these tests as part of their routine care.

Clinical scoring systems including Child–Pugh Score and Barcelona Clinic Liver Cancer (BCLC) stage were also calculated. The number of patients for whom the Child–Pugh score could be calculated was limited by the inability of patients to pay for the international normalized ratio (INR) test. Patients who were available were contacted and the test paid for through research funds, in order to derive the score. The aspartate aminotransferase (AST)-to-platelet ratio index (APRI) score, which is a non-invasive test to estimate hepatic fibrosis and cirrhosis using the platelet count and AST level, was measured in all patients. The World Health Organization (WHO) recommends a single high cut-off APRI score of > 2 for the identification of cirrhosis in patients at risk. NAFLD was diagnosed clinically if serologies were negative, there was no significant history of alcohol consumption, and patients had one or more risk factors such as type 2 diabetes and obesity.

Radiological investigations obtained were findings from abdominal ultrasonography (US) including liver size and appearance, number and size of nodules in the case of malignancy, and additional information including tumor invasion and metastasis. Abdominal US was performed in all patients. Socio-demographic information, psycho-social, and dietary history were recorded. Clinical data (history and physical examination) were obtained by the treating physicians, and additional questionnaire data were obtained by trained research assistants. Data was collected in hard copy and subsequently entered into an online database designed at the Department of Medical Epidemiology and Biostatistics, Karolinska Institutet, Sweden.

### Statistical analysis

Where relevant, patients were grouped into their main diagnosis of HCC, liver cirrhosis or chronic HBV. Two sample t-test or ANOVA was used to compare differences between continuous variables. Mann–Whitney and Kruskal Wallis tests were used to compare differences between medians, and Pearson’s chi square test was used to determine differences between categorical variables. Univariate and multivariate logistic regression were used to determine sensitivities and specificities, and to examine the association of clinical characteristics with severity of disease at presentation, with adjustment for confounders. For missing data, available case analysis was performed. Performance of the APRI score in diagnosis of cirrhosis and the AFP level in the diagnosis of any stage and early stage HCC was determined using AUROC analysis. Stata version 15 (StataCorp) was used to carry out all analyses. A *p* value of < 0.05 was determined as statistically significant.

## Results

A total of 141 liver cancer, 216 liver cirrhosis, and 218 chronic HBV patients were recruited during the study period. Fifty three percent (53%) of HCC patients developed HCC on a background of cirrhosis. The patient groups had no significant differences in demographic profile, except for age, where chronic HBV patients were younger (median age 35 years, *p* value < 0.001) (Table 1). There were more male than female patients in both the HCC and cirrhosis groups, with male to female ratios of 3.2:1 and 2.3:1, respectively. The overall median age at diagnosis was 44 years (IQR 36–54) for HCC patients and 46 years (IQR 37–46) for cirrhotics, and there was no significant difference in age at diagnosis between the two groups of ESLD patients (*p* value 0.4). Patients with HCV-associated disease had an older age at diagnosis—median age 53 for HCC (*p* value 0.03) and 49 for cirrhosis (*p* value 0.16) compared with HBV-infected persons. Monthly household income of most patients was less than 1000 Ghana cedis (GHS) or 182 US Dollars (USD), for 73–77% of patients in all 3 groups.

**Table 1:** Sociodemographic and laboratory information of ESLD and Chronic HBV patients

	HCC n/N* (%)	Cirrhosis n/N* (%)	Chronic HBV n/N* (%)
Sociodemographic information			
Sex			
Men	106/139 (76.3)	150/214 (70.1)	125/218 (57.3)
Women	33/139 (23.7)	64/214 (29.9)	92/218 (42.2)
Age at diagnosis, median (IQR)			
Overall	44 (36–54)	46 (37–46)	35 (28–44)
HBV	43 (36–48)	42 (34–50)	–
HCV	53 (47–65)	49 (40.5–59.5)	–
ALD	42 (39–50)	51 (44–60)	–
Monthly household income in Ghana cedis, (GHS) with USD ($) equivalent			
< 500 ($90)	44/100 (44.0)	91/151 (60.3)	88/165 (53.3)
500–999 ($91–182)	29/100 (29.0)	24/151 (15.9)	39/165 (23.6)
1000–2499 ($182–454)	24/100 (24.0)	27/151 (17.9)	32/165 (19.4)
> 2500 (> $454)	3/100 (3.0)	9/151 (6.0)	6/165 (3.6)
Laboratory information			
Platelet, mean (SD)	224.9 (150)	141.3 (104)	221.1 (59)
Prothrombin time INR, mean (SD)	1.3 (0.7)	1.8 (1.3)	–
Albumin (g/L), mean (SD)	35.1 (11.9)	30.8 (9.9)	42.0 (7.0)
APRI score, median (IQR)	1.5 (0.7–2.9)	1.4 (0.7–3.0)	0.3 (0.3–0.5)
AFP (ng/mL), median (IQR)	528.1 (31.45–3149)	4.6 (2.6–8.7)	–
HBV DNA (IU/mL), median (IQR)	21,615 (8580–122,500)	1903.5 (20–76,399)	3503 (489–15,300)

n/N* The total number of patients was 141 HCC, 216 liver cirrhosis and 218 chronic HBV, however not all questions were answered by all participants

Missing laboratory data: Platelet count (HCC 36, cirrhosis 50, and chronic HBV 117); INR (HCC 59 and cirrhosis 103); Albumin (HCC 33, cirrhosis 47, and chronic HBV 99); AFP (HCC 80 and cirrhosis 147); HBV DNA (HCC 86, cirrhosis 89, and HBV 132)

There were significant differences in the laboratory test results at presentation between patient groups (Table 1). Mean platelet count at presentation fell within the normal range (150–400 × 10^9^/L) for liver cancer patients, but was significantly lower among cirrhosis patients (*p* value < 0.001). Similarly, the mean albumin level at presentation was low or low normal among ESLD patients (normal range 35–50 g/L), although significantly worse among cirrhotics (*p* value 0.001), and was normal for chronic HBV patients. The median APRI scores for HCC and cirrhosis patients were 1.5 and 1.4, respectively, below the WHO recommended high cut-off of > 2, with an IQR of 0.7–3.0 for patients with cirrhosis. Indeed, only 43.0% of HCC patients with cirrhosis, and 38.4% of cirrhosis patients without HCC had an APRI score above 2. Among all patients who tested HBsAg positive, the sensitivity of APRI for the diagnosis of cirrhosis was 45.4% when the cut-off > 2 was used (Table 2), and there was remarkably improved sensitivity, without much loss to specificity when a cut-off of > 1 was applied.

**Table 2:** Sensitivity and specificity of APRI in diagnosis of cirrhosis in HBV patients

APRI cut-off	Sensitivity (%)	Specificity (%)
0.67	84	86
1	75.9	89
2	45.4	95

Regarding the cost of care, only one third of patients with liver cirrhosis and less than half of HCC patients were able to afford testing for AFP. Similarly, very few ESLD patients who were HBsAg positive were able to afford testing for HBV DNA levels; 12/98 (12.2%) for HCC and 14/102 (13.7%) for cirrhosis. Of the HBsAg positive patients able to afford HBV DNA testing, only 7/12 (58%) of the HCC patients and 2/12 (16.7%) of the cirrhosis patients would have qualified for antiviral therapy based on their alanine aminotransferase (ALT) and HBV DNA levels if their HCC or cirrhosis status were unknown, using the current WHO treatment guidelines.

**Fig. 2:**
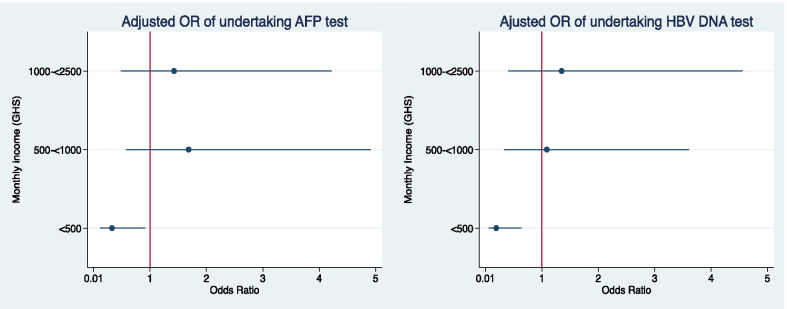
Age adjusted odds ratio of undertaking AFP and HBV DNA test

There was an association between monthly income and the ability to undertake HBV DNA and AFP testing, which patients must currently pay for out of pocket, irrespective of national health insurance status. Individuals who had a monthly income of less than GHC 500 (< $91) had lower odds of undertaking either of these tests compared with those who had a monthly income of more than GHC 2500, after adjusting for age (Fig. 2).

**Fig. 3:**
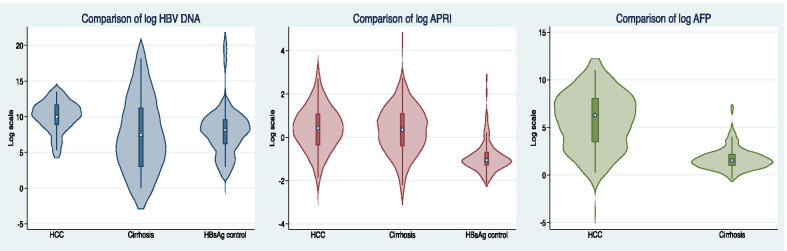
Comparison of HBV DNA, APRI Score and AFP among HCC, cirrhosis and chronic HBV patients

**Fig. 4:**
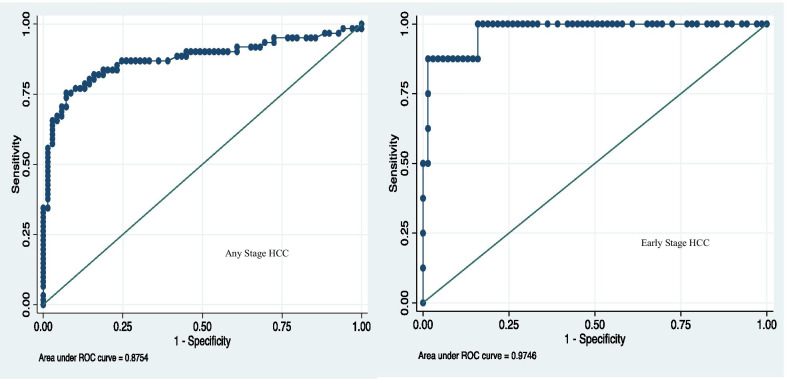
ROC curve of AFP for the diagnosis of hepatocellular carcinoma

Although there was substantial data missing in the analysis for differences between groups with regard to AFP and HBV DNA levels (Table 1), some disparities could still be noted. The median HBV DNA levels were significantly lower among cirrhotics compared with HCC patients (Fig. 3). The median HBV DNA was also lower for chronic HBV patients compared with HCC patients, though some of these patients were on antiviral therapy. The median AFP was higher in HCC patients than in cirrhotics without HCC. The performance of AFP in distinguishing patients with HCC from liver cirrhosis patients was assessed using receiver-operating characteristic (ROC) curves (Fig. 4). The area under the curve (AUC) for all HCC patients compared with non-HCC patients was 0.88 (0.81–0.94); for very early or early stage HCC (BCLC 0 and BCLC A) patients compared with non-HCC patients, the AUC was 0.97 (95% CI 0.93–1.00). It is noted however that there were only 8 patients with BCLC 0 and A with large single tumours, on which performance of AFP was performed, which could account for some diagnostic bias.

The optimum AFP value for the diagnosis of HCC using the Youden index was 29.5 ng/ml, which yielded a sensitivity of 75.4% and specificity of 91.3% (Table 3). The sensitivities and specificities for various AFP cut-offs are shown in Table 3. The recommended diagnostic AFP value of 400, yielded a sensitivity and specificity of 52.5% and 98.6% respectively in the diagnosis of any stage HCC, and 87.5% and 98.6% respectively in the diagnosis of early HCC in this cohort. A cut-off of the upper limit of normal for AFP (7 ng/ml), yielded higher sensitivity but relatively lower specificity (68.1%) in the determination of HCC.

**Table 3:** Sensitivity and specificity of AFP values in diagnosis of hepatocellular carcinoma

AFP (ng/ml)	Any stage HCC		Early stage HCC (BCLC 0 and A)^a^	
	Sensitivity (%)	Specificity (%)	Sensitivity (%)	Specificity (%)
7.0	86.9	68.1	100	68.1
29.5	75.4	91.3	87.5	91.3
116.2	57.4	97.1	87.5	97.1
400	52.5	98.6	87.5	98.6

^a^8 patients with BCLC stage 0 or stage A

The common presenting symptoms and significant examination and clinical grading scores for the participating patients are shown in Table 4. Abdominal pain and anorexia were more prominent symptoms among HCC patients. The majority of ESLD patients subjectively described substantial weight loss (89.8% of HCC and 84.3% of liver cirrhosis patients). There was a significantly higher odds of presenting as Child–Pugh class B in patients who reported a history of weight loss [Odds ratio (OR) 5.9, 95% confidence interval (CI) 1.4–25.1] (Table 6). Furthermore, a higher proportion of patients with liver cirrhosis presented with ascites (72% with any ascites) compared to those with HCC (54.8% with any ascites; *p* value 0.001). Child–Pugh class of B or C was more common than class A in both HCC and cirrhosis patients (*p* value 0.01), being present in 62.4% of cirrhotic HCC patients and 79.5% of cirrhosis patients. Notably, approximately twice the proportion of cirrhotic patients (37.3%) versus HCC patients (15.6%) were classified as Child–Pugh class C.

**Table 4:** Symptoms, classification, and risk factors of ESLD patients at presentation

	HCC n/N^a^ (%)	Cirrhosis n/N^a^ (%)	*p* value
Abdominal pain or discomfort	110/129 (85.2)	122/204 (59.8)	< 0.001
Jaundice	38/128 (29.7)	59/204 (28.9)	0.88
Weight loss	115/128 (89.8)	172/204 (84.3)	0.15
Anorexia	67/128 (52.3)	64/205 (31.2)	< 0.001
Fever	61/126 (48.4)	85/203 (41.9)	0.25
Ascites (clinically determined)			
None	60/133 (45.1)	57/203 (28.1)	0.001
Mild	24/133 (18.0)	60/203 (29.6)	0.02
Moderate–Severe	49/133 (36.8)	86/203 (42.4)	0.3
Hepatic encephalopathy			
None	118/129 (91.5)	194/202 (96.0)	0.08
Grade 1–2	11/129 (8.5)	8/202 (4.0)	0.08
Child–Pugh Class			
A	12/32 (37.5)	17/83 (20.5)	0.01
B	15/32 (46.8)	35/83 (42.2)	0.9
C	5/32 (15.6%)	31/83 (37.3)	0.01
Risk factors			
HBV	98 (69.5)	102 (47.2)	< 0.001
HCV	9 (6.4)	8 (3.7)	0.3
Alcohol	15 (10.6)	63 (29.2)	< 0.001
Autoimmune	1 (0.7)	4 (1.9)	0.3
NAFLD	0 (0)	3 (1.4)	0.1
Unknown^b^	22 (15.6)	40 (18.5)	0.4

^**a**^The total number of patients was 141 HCC, 216 liver cirrhosis and 218 chronic HBV, however not all questions were answered by all participants

^b^Unknown after testing for viral hepatitis and without history suggestive of other causes

The risk factors for ESLD for recruited patients were obtained by review of their medical records based on available clinical, laboratory and radiological data. No patients were documented to have undergone a liver biopsy, likely because of the cost of the procedure. The most common risk factor for ESLD was hepatitis B infection (Table 4) accounting for 69.5% of HCC cases and 47.2% of cirrhosis cases. Hepatitis C infection was attributed with 6.4% of HCC cases and 3.7% of cirrhosis cases. Four patients with HCC and 4 patients with liver cirrhosis had HBV-HCV co-infection. None of the patients recruited tested positive for antibodies to HIV-1 or HIV-2 upon serological testing using enzyme linked immunosorbent assay (ELISA) test-kits. Alcohol was associated with a higher proportion of cirrhosis than HCC (29.2% vs. 10.6%; *p* value < 0.001). Less common risk factors for cirrhosis were NAFLD (1.4%) and autoimmune hepatitis (1.9%). Almost a fifth of all ESLD patients had no known underlying liver disease.

Of the 81 HCC patients for whom BCLC stage could be determined (Table 5), only one patient presented with very early stage disease (BCLC 0) and 11.1% presented with early stage BCLC A disease. The majority of patients presented with intermediate (19.8%), advanced (50.6%) or terminal stage (17.3%) disease. Although 39.5% of HCC patients presented with a single nodule, most had tumors that were larger than 3 cm. The mean tumor size in patients presenting with a single nodule was 9.4 cm. Radiological information was obtained via abdominal ultrasound performed by radiographers. Patients with a single nodule were classified as beyond BCLC stage A if their performance status was 2 or greater, or the Child–Pugh class was C (Table 6).

**Table 5:** Staging and characteristics of HCC

	HCC cases	
BCLC stage	n = 81^b^	%
0	1	1.2
A	9	11.1
B	16	19.8
C	41	50.6
D	14	17.3
Tumor features		
Single nodule	32	39.5
Multinodular	49	60.5
Smallest tumor size (cm)	1.2	–
Largest tumor size (cm)	19	–
Mean tumor size (mean, SD) (single nodule)	9.4 ± 4.2	–

^a^BCLC was determined for 81 HCC cases. Some patients did not have INR measurement, but could be classified because of Child-Pugh score of > 10 (class C) even assuming a normal INR

**Table 6.** Multinomial regression analysis investigating correlates of presenting as Child–Pugh A versus Child–Pugh B or C

	Child–Pugh B		Child–Pugh C	
	Versus		Versus	
	Child–Pugh A		Child–Pugh A	
	OR	95% CI	OR	95% CI
Weight loss	5.9	1.4–25.1	3.1^a^	0.7–14.4
Albumin	0.8	0.8–0.9	0.8	0.7–0.9
APRI score	1.2^a^	0.9–1.5	1.3	1.1–1.8
Male sex	0.8^a^	0.3–1.8	1.0^a^	0.3–2.5

^a^Not significant

## Discussion

The findings of our study demonstrate the relatively early age at presentation for patients with both liver cancer and cirrhosis as well as the unequal sex distribution for both conditions in Ghana. We describe the performance of APRI score and AFP in ESLD and identify financial constraints as a potential factor resulting in insufficient diagnostic workup. We additionally report the clinical stages at presentation, providing evidence that these patients mostly present with late stage disease. Furthermore, our study describes the causes of ESLD in Ghana, highlighting the disease burden of hepatitis B virus infection.

The median age at diagnosis of 44 years for liver cancer among study participants is consistent with findings from previous studies in which it has been demonstrated that HCC patients in sub-Saharan Africa present at a younger age than patients in other parts of the world [9, 12]. This has been attributed in part to the difference in etiology, in that hepatitis C and alcoholic liver disease are more frequent causes of liver cancer in Europe and the Americas, compared with sub-Saharan Africa. That being said, in China where similar to Ghana, the main cause of HCC is HBV infection, the age at presentation is still higher [13]. It has been suggested that differences in oncogenicity of the prevailing HBV genotypes in sub-Saharan Africa, coupled with host genetic and environmental factors such as dietary exposure to aflatoxin, could play a role in the progression of disease, and contribute to the differences in age at onset of HCC. Additionally, there is growing evidence of the role of the gut microbiome in liver disease progression, and the microbiome is known to vary across ethnicities owing to several factors including diet, lifestyle, and socioeconomics [14]. Studies related to the gut microbiome in ESLD have largely been conducted in the developed world, and it would be useful to explore similarities and differences in the microbiomes of ESLD patients in an African cohort compared with those from the Western world, in an attempt to better understand liver disease dynamics.

The median age at diagnosis for cirrhotic patients was 46 years, similar to the findings of a single-center study conducted at the Korle Bu Teaching Hospital in Accra, Ghana, in which the mean age of patients was 45 years [11]. This figure has not changed significantly in almost 15 years, confirming that cirrhosis affects individuals during their most productive years of life [15]. Although the economic impact of the burden of disease has not been fully enumerated in Ghana, there is evidence from countries in which preventive and treatment strategies do exist, that there is a high economic burden and significant loss of productivity associated with the diagnosis of liver cirrhosis [16]. Other studies in Asia and the Americas demonstrate a mean age at diagnosis of cirrhosis of about 60 years, over a decade older than observed in Ghana [17, 18].

The higher male to female ratio was not surprising, as it has been consistently demonstrated that this gender disparity exists, particularly for HCC. It is unlikely that health seeking behavior of patients accounts for this gender disparity, as women more readily utilize outpatient services in Ghana [19]. Of the few studies that have investigated why more males than females develop HCC, one study described the influence of estrogen in reducing interleukin-6 and subsequently reducing tumorigenesis [20], whilst a more recent study suggested that the cause might be related to the fact that men produce lower levels of the hormone adiponectin [21].

The median APRI scores among HCC and cirrhotic patients were 1.5 and 1.4 respectively, both below the recommended cutoff value of 2 for non-invasive diagnosis of cirrhosis among adults in low resource settings. Moreover, this cut-off demonstrated low sensitivity for diagnosing cirrhosis in our cohort. This is important because cirrhosis is one of the indications for starting antiviral treatment in patients with chronic hepatitis B infection. The rationale for the relatively high cut-off value is that using a low cut-off would result in a larger number of false positives, and that patients in need of antiviral therapy who have an APRI of < 2 would likely fulfil other eligibility criteria such as high ALT or HBV DNA levels [22]. However, as demonstrated in our results, the majority of patients were unable to afford HBV DNA testing, and relying on this result as the cue for initiating therapy when APRI is < 2 may result in missing opportunities for treatment. Additional studies validating the APRI score and other non-invasive tests of fibrosis and cirrhosis in Ghana and other sub-Saharan African countries are warranted, since only one sub-Saharan African country was included in the validation of APRI and the subsequent recommendations. Furthermore, studies to identify the optimal criteria for initiation of antiviral treatment for chronic hepatitis B infection in sub-Saharan Africa are needed.

The cost of tests was a notable barrier to optimal care. The majority of patients reported a household income of less than GHC 1000 ($182). Putting this into context, a patient would typically spend GHC 650 ($118) or more on currently recommended tests such as HBV DNA, AFP, and INR alone, irrespective of whether they are enrolled in the National Health Insurance Scheme in Ghana. It is known that inability to afford diagnostic tests hinders not only clinical management, but also the ability of public health systems to adequately assess and characterize the burden of disease in low- and middle-income countries [23]. AFP performed well in distinguishing HCC from cirrhosis, thus ensuring the test is made affordable for patients could potentially increase HCC surveillance among at-risk patients in Ghana.

Hepatitis B infection was the primary risk factor for ESLD among patients in Ghana, reflecting the high burden of chronic hepatitis B infection in Ghana. The national prevalence of HBV infection is 12.3% [24], demonstrating high endemicity in the country. Efforts to reduce HBV prevalence have focused on immunization of infants against hepatitis B since the year 2002, however there remains a large older cohort of individuals who did not receive vaccination, and are therefore still at risk of development of HBV-related end-stage liver disease. Additionally, the threat of mother to child transmission still exists, since birth-dose vaccination and immunoglobulin administration for newborns are still not routinely practiced in Ghana. Strategies must be developed to manage these challenges in order to reduce the morbidity of HBV infection and ESLD in the region.

The sero-prevalences of antibodies to HCV were 3.7% among persons with cirrhosis and 6.4% among those with HCC. Other studies have reported estimates between 2.7 and 8.7% for cirrhosis and 6% for HCC [9, 11, 25]. Although the prevalence of HCV-related liver disease is lower than in other countries such as Egypt and the United States, HCV treatment is not readily available in Ghana, and access to treatment is further hindered by the need for expensive tests, including HCV viral load and genotyping. Public health advocacy and education are therefore still necessary to reduce the threat of HCV infection in Ghana.

Both cirrhotic and HCC patients presented with advanced disease. Sixty percent of HCC cases had multinodular lesions. This is not an uncommon finding in sub-Saharan African countries. Yang et al. [9] in 2017 reported that 84% of liver cancer cases presented with multinodular disease. In Ghana, Gyedu et al. [10] found that only 8% of persons with HCC seen at the Komfo Anokye Teaching Hospital in Accra between 2007 and 2013 were eligible for curative treatment. In our study, the AFP performed well as a diagnostic test in the detection in HCC. This highlights the urgent efforts needed to develop strategies that will improve liver cancer surveillance in Ghana, in order to enhance eligibility for curative treatment and reduce mortality.

This is also true with respect to cirrhosis surveillance in patients with known risk factors, because our results showed that cirrhotics predominantly presented with Child–Pugh class B and C. Furthermore, high APRI score and weight loss were significantly associated with a high Child–Pugh score at presentation. In Ghana, a lack of nutritional assessment and local nutritional guidelines were found to contribute to poor nutritional management of cirrhosis patients [26], and this may potentially contribute to the high proportion of patients with weight loss. For patients who may not be able to perform necessary diagnostic tests, surrogates such as weight loss and high APRI may be useful in understanding the degree of morbidity and the risk of mortality, although more studies are needed in this regard in Ghana. The causes of weight loss and sarcopenia in ESLD are multifactorial and include reduced synthesis of glycogen, increased protein breakdown, malnutrition and other factors [27]. Treatment is therefore multifaceted, and pharmacologic and nutritional management are important for patients presenting with significant weight loss.

## Limitations

One of the major limitations of the study was the inability of patients to afford particular diagnostic tests, which limited the derivation of clinical scoring for severity of Child–Pugh and BCLC scores. To elaborate, this study relied on abdominal ultrasound for diagnosis of cirrhosis, since elastography is not yet performed in Ghana, and patients must pay for liver biopsies out of pocket. As a result, it is possible that patients with Child–Pugh Class A cirrhosis were underreported. Likewise, due to financial constraints, the majority of patients could not afford abdominal CT scans with intravenous contrast, therefore clinicians relied on abdominal ultrasonography, which has lower sensitivity and specificity, for the diagnosis of HCC. It is therefore possible that cases of early HCC were less readily identified. A further limitation in measuring AFP levels is the fact that patients receiving HBV therapy were not captured, and it is known that HBV therapy may reduce AFP levels in the bloodstream.

Additional limitations to this study include the fact that patients were recruited from the teaching hospital outpatient departments, thus inpatient ESLD patients, who typically have higher morbidity, were not included in the study. It is therefore possible that the overall disease severity of patients with ESLD is worse than we observed here. Furthermore, no patients with Grade 3 encephalopathy or higher were recruited because they were clinically unstable, and this would also have affected our ability to comprehensively ascertain the clinical characteristics of the patient cohorts; however, the study provides an accurate representation of the burden of liver disease seen in the ambulatory outpatient setting. Finally, this study was conducted at public teaching hospitals in Ghana. It is possible that patients who seek traditional and alternative medicine (TAM) are not accounted for in this study, which may cause some selection bias, however it is important to add that symptomatic ESLD patients are also referred from these facilities once non-allopathic care is not successful.

## Conclusions

This study suggests that ESLD patients in Ghana may present at an early age and potentially with significantly advanced disease. It also shows a higher male preponderance, and reflects the challenges in effecting optimal diagnostic and treatment algorithms due to the inability of many patients to afford the cost of standard care. Our results draw attention to the importance of efforts to reduce the cost of evaluation and imaging studies, including coverage for liver disease care in National Health Insurance programs and the need for improved surveillance for ESLD in patients with known risk factors in order to reduce the disease burden and improve the outcomes of patients with ESLD in Ghana.

## Abbreviations

AFP: Alpha fetoprotein
ALT: Alanine aminotransferase
APRI: Aspartate aminotransferase to platelet ratio index
AST: Aspartate aminotransferase
BCLC: Barcelona clinic liver cancer
ESLD: End-stage liver disease
GHS: Ghana cedi
HBsAg: Hepatitis B surface antigen
HBV: Hepatitis B virus
HCC: Hepatocellular carcinoma
HCV: Hepatitis C virus
HIV: Human immunodeficiency virus
INR: International normalized ratio
NAFLD: Non-alcoholic fatty liver disease
TAM: Traditional and alternative medicine
USD: United States Dollar
US: Ultrasound
WHO: World Health Organization

## Availability of data materials

Data generated by and used in the study is available from the corresponding author upon reasonable request.
